# Kidney Ischemia-Reperfusion Decreases Hydrogen Sulfide and Increases Oxidative Stress in the Heart

**DOI:** 10.3390/biom10111565

**Published:** 2020-11-17

**Authors:** Charith U. B. Wijerathne, Susara Madduma Hewage, Yaw L. Siow, Karmin O

**Affiliations:** 1 St. Boniface Hospital Research Centre, Winnipeg, MB R2H 2A6, Canada; CWijerathne@sbrc.ca (C.U.B.W.); smaddumahewage@sbrc.ca (S.M.H.); csiow@sbrc.ca (Y.L.S.); 2 Department of Animal Science, University of Manitoba, Winnipeg, MB R3T 2N2, Canada; 3 Department of Physiology & Pathophysiology, University of Manitoba, Winnipeg, MB R3E 0J9, Canada; 4 Agriculture and Agri-Food Canada, St. Boniface Hospital Research Centre, Winnipeg, MB R2H 2A6, Canada

**Keywords:** hydrogen sulfide, oxidative stress, acute kidney injury, heart, glutathione

## Abstract

Patients with acute kidney injury (AKI) have an increased risk of cardiovascular disease. The underlying mechanism of AKI-induced heart injury is not well-understood. Hydrogen sulfide (H_2_S), at physiological concentrations, has been implicated in cardiovascular protection through redox balance and vessel relaxation. Cystathionine gamma-lyase (CSE) plays an essential role in H_2_S production in the heart. The present study investigated the effect of AKI on H_2_S production and oxidative stress in the heart. AKI was induced by kidney ischemia-reperfusion in male and female Sprague-Dawley rats, which led to an increase in plasma creatinine and blood urea nitrogen levels. There was a significant increase in lipid peroxidation and a decrease in glutathione (antioxidant) levels in the plasma and heart, indicating systemic and cardiac oxidative stress. Kidney ischemia-reperfusion reduced CSE expression and H_2_S production in the heart. There was a decrease in antioxidant transcription factor Nrf2 level in the nucleus and an increase in inflammatory cytokine (IL-6, TNF-α) expression in the heart. These results suggest that AKI can down-regulate CSE-mediated H_2_S production, reduce glutathione levels and increase oxidative stress in the heart. This may contribute to an increased risk of cardiovascular disease in AKI.

## 1. Introduction

Acute kidney injury (AKI) is a common clinical condition in critical care settings, characterized by a decline in kidney function, over a short period of time. AKI, not only causes kidney damage, but can also injure other organs, such as the heart, lungs, liver, intestines, and the brain [1,2]. The risk of cardiovascular disease is significantly increased in patients with kidney disease [3]. Cardiovascular disease represents a main cause of death in AKI patients [4,5]. However, our understanding of the molecular pathways that lead to cardiovascular injury and the potential targets for cardiovascular protection in patients with kidney disease is limited. Kidney ischemia-reperfusion injury due to surgical procedures, kidney transplantation or in critically ill patients is one of the most common causes for AKI. Ischemia-reperfusion triggers a series of biochemical and molecular changes that can elicit oxidative stress, inflammation and apoptosis, leading to injuries in the kidneys as well as in the other organs [1,6].

Oxidative stress and inflammation are pathological features in cardiovascular diseases, including myocardial infarction, heart failure, hypertension, and atherosclerosis. Oxidative stress arises when the surplus of reactive oxygen species (ROS) overwhelms the detoxifying capability of antioxidant defense, leading to an accumulation of ROS [7,8]. Under physiological conditions, the endogenous antioxidant defense system (enzymes and non-enzymatic molecules) functions efficiently to reduce ROS accumulation in the body [8]. Glutathione, a tripeptide, is the major endogenously synthesized non-enzymatic antioxidant that can scavenge ROS. It is synthesized by glutamate-cysteine ligase (catalytic subunit Gclc, modifier subunit Gclm) and glutathione synthetase [9,10]. The reduced form of glutathione (GSH) exists in an abundant amount in the heart and plays an important role against oxidative stress [10]. The depletion of GSH is linked to increased risk of cardiovascular diseases such as myocardial infarction, atherosclerosis, and hypertension [10].

Hydrogen sulfide (H_2_S) and its derivatives (persulfides, polysulfides) have emerged as biologically active compounds in human health and disease [11,12,13]. At physiological concentrations, H_2_S serves as a vasodilator and a neurotransmitter [11,12]. The cardiovascular protective effect of H_2_S is attributed to its antioxidant, anti-inflammatory and anti-apoptotic properties against myocardial ischemia-reperfusion injury, myocardial infarction, cardiac arrhythmia and hypertrophy [12,14,15]. The supplementation of exogenous H_2_S donors offers protection against myocardial ischemia-reperfusion injury [15]. The synthesis of H_2_S is tightly regulated under physiological conditions. Cystathionine-β-synthase (CBS) and cystathionine-γ-lyase (CSE) are the main enzymes responsible for endogenous H_2_S synthesis [11]. Impaired endogenous H_2_S synthesis is associated with various disorders ranging from cardiovascular disease, kidney disease, metabolic disease to stroke [12,16]. CSE-mediated H_2_S synthesis is the major source of H_2_S in the cardiovascular system [15]. Mice that were deficient in CSE displayed an increase in oxidative stress and myocardial injury [17]. On the other hand, the injection of exogenous H_2_S donors could improve cardiac function [18]. The antioxidant action of H_2_S is attributed, in part, to increased production of GSH and activation of nuclear factor erythroid 2-related factor 2 (Nrf2) [19]. The depletion of H_2_S was associated with low GSH production, Nrf2 expression and ROS accumulation [19]. Nrf2 is a key transcription factor that plays an important role in anti-oxidative stress and has cardiac protection through the upregulation of gene expression of enzymes involved in antioxidant defense [19,20]. Downregulation of Nrf2 pathway was reported in atherosclerosis, myocardial infarction and heart failure [21,22,23]. Activation of the Nrf2 signaling pathway has become an attractive target for the treatment of cardiovascular disease.

Despite recent advancement in dialysis and kidney transplantation, the mortality among patients with AKI complicated by multi-organ dysfunction remains high (estimated to be 50%) [24]. Cardiovascular disease still represents a main cause of death in AKI patients. However, the underlying mechanisms are not well understood. In the present study, we investigated the effect of AKI on cardiac H_2_S production and oxidative stress in male and female rats with kidney ischemia-reperfusion injury.

## 2. Materials and Methods

### 2.1. Kidney Ischemia-Reperfusion

Sprague-Dawley rats were randomly divided into 4 groups: male kidney ischemia-reperfusion, male control, female kidney ischemia-reperfusion and female control. Each group consisted of 6 rats. Kidney ischemia was induced in male and female rats (250–300 g) by clamping the left renal pedicle with a non-traumatic vascular clamp for 45 min, as described in our previous studies [25,26,27,28]. At the end of ischemia, the clamp was removed to allow blood flow to the left kidney (reperfusion) and right nephrectomy was performed. As a control (sham-operated), male and female rats were subjected to the same surgical procedure but without kidney ischemia. The heart rate was measured in rats using the CODA™ mouse rat tail-cuff system (Kent Scientific corporation, Torrington, CT, USA). Rats were sacrificed 24 h after the surgery. All procedures were performed in accordance with the Guide to the Care and Use of Experimental Animals published by the Canadian Council on Animal Care and approved by the University of Manitoba Protocol Management and Review Committee. Blood was collected from the abdominal aorta and plasma was prepared by centrifugation at 3000× *g* for 20 min at 4 °C. Plasma and heart were stored at −80 °C until analysis.

### 2.2. Biochemical Analysis

Kidney function was examined by measuring plasma creatinine and blood urea nitrogen (BUN) using the Cobas c111 analyzer (Roche, Risch-Rotkreuz, Switzerland). The malondialdehyde (MDA) in plasma and heart tissue was determined by using the thiobarbituric acid reactive substances (TBARS) assay [25,29]. Increased MDA was used as a biomarker indicative of oxidative stress. For the measurement of GSH, an aliquot of heart tissue homogenate (in 5% sulfosalicylic acid) or plasma was added to 0.2 M phosphate buffer containing 0.01 M 5,5’-dithiobis-(2-nitrobenzoic acid) (DTNB). The absorbance was measured at 412 nm after incubation for 15 min at room temperature [29]. For the measurement of H_2_S, an aliquot of heart homogenate was added to a reaction mixture-A containing L-cysteine (10 mM), homocysteine (10 mM), pyridoxal 5’-phosphate (2 mM) and S-Adenosyl-L-methionine (0.05 mM) in an Erlenmeyer flask saturated with N_2_ gas. After incubation at 37 °C for 30 min, a filter paper saturated with 1% zinc acetate and 12% NaOH was added to entrap H_2_S. The entrapped H_2_S in the filter paper was then dissolved in a reaction mixture-B containing 20 mM N,N-dimethyl-p-phenylenediamine sulphate and 30 mM FeCl_3_. The reaction was allowed to proceed for 10 min in dark and the absorbance was measured at 670 nm [26,27,29,30]. Plasma samples were directly reacted with reaction mixture-B [27,29].

### 2.3. Histological Examination

For histological examination, the heart was immersion fixed in 10% neutral-buffered formalin followed by embedding in paraffin. The paraffin-embedded cross sections (5 µm) were prepared and stained with hematoxylin and eosin (H and E) to examine histological changes in the heart tissue. The images were captured by using Olympus BX43 Upright Light Microscope with an Olympus Q-Color3 digital camera (Olympus Corporation, Tokyo, Japan) and analyzed by using Image-Pro Plus 7.0 (Media Cybernetics, Bethesda, MD, USA).

### 2.4. Western Immunoblotting Analysis

The protein levels of glutathione synthesizing enzymes and H_2_S synthesizing enzymes were determined by Western immunoblotting analysis. In brief, the heart was homogenized in a buffer containing 20 mM Tris pH 7.4, 150 mM NaCl, 1 mM EGTA, 1 mM EDTA, 2.5 mM sodium pyrophosphate, 1 mM β-glycerophosphate, 1 mM sodium orthovanadate, 2.1 µM leupeptin, 1 mM PMSF, and 1% (*v/v*) Triton X-100 [31]. After centrifugation at 3000× *g* for 20 min, proteins in the supernatant were collected and quantified by Bradford protein assay. The isolated proteins were placed in a buffer (0.5 M Tris–HCl pH 6.8, 10% glycerol, 2% (*w/v*) SDS, 5% (*v/v*) β-mercaptoethanol, and 0.05% bromophenol blue) and denatured by boiling at 95–100 °C for 5 min. An aliquot of total proteins (10–50 µg) was applied to a sodium dodecyl sulfate (SDS) 12% polyacrylamide gel. After electrophoresis, proteins in the gel were transferred to a nitrocellulose membrane. The membrane was probed with one of the following primary antibodies: Rabbit anti-Gclc monoclonal antibody (1:1000), rabbit anti-Gclm monoclonal antibody (1:1000), rabbit anti-glutathione synthetase (GS) monoclonal antibody (1:1000), rabbit anti-CSE monoclonal antibody (1:3000) and rabbit anti-CBS monoclonal antibody (1:5000). The anti-Gclc, anti-Gclm, and anti-GS monoclonal antibodies were purchased from Abcam, Cambridge, United Kingdom while the anti-CSE and anti-CBS monoclonal antibodies were purchased from Genetex, Irvine, CA, USA. The membrane was then incubated with HRP conjugated anti-rabbit IgG secondary antibodies (1:2000 or 1:5000, Cell Signalling Technology, Danvers, MA, USA). To ensure equal protein loading, the same membrane was probed with rabbit anti-GAPDH primary antibodies (1:5000, Cell Signalling Technology). Nuclear proteins were prepared as described in our previous study [29]. In brief, a portion of the heart was homogenized in Tris-buffered saline followed by centrifugation at 3000× *g* for 20 min. The pellet was dissolved in buffer A containing 10 mM HEPES, 10 mM KCl, 0.1 mM EDTA, 0.1 mM EGTA, protease inhibitors, and 10% Nonidet P-40 and centrifuged at 15,000× *g* for 15 min. The resulting nuclear pellet was dissolved in buffer B containing 20 mM HEPES, 0.4 M NaCl, 1 mM EDTA, 1 mM EGTA, and protease inhibitors. The nuclear proteins (100 µg/well) were separated by SDS 8% polyacrylamide gel electrophoresis. Nrf2 protein was identified by using rabbit anti-Nrf2 monoclonal antibodies (1:500, Abcam) and HRP conjugated anti-rabbit IgG secondary antibodies (1:3000). To ensure equal loading of nuclear proteins, the same membrane was probed with rabbit anti-lamin B1 (nuclear envelop marker) polyclonal primary antibodies (1:500, Abcam). The protein bands were visualized by using ECL detection system (Millipore Ltd., Burlington, MA, USA). The relative intensity of each protein was quantified by using Bio-Rad Quantity One software version 4.6.8 for Windows (Bio-Rad, Hercules, CA, USA).

### 2.5. Real-Time PCR Analysis

The mRNA expression of glutathione synthesizing enzymes, H_2_S synthesizing enzymes, *Nrf2* and inflammatory cytokines (*TNF-α* and *IL-6*) in the heart was determined by using a StepOnePlus™ Real-Time PCR System (Applied Biosystems, Foster City, CA, USA). In brief, total RNA was prepared from the heart using TRIzol reagent (Thermo Fisher Scientific, Waltham, MA, USA). Total RNA (1–2 µg) was converted to cDNA through reverse transcription. A real-time PCR reaction mixture was prepared which consisted of 2 µL of cDNA product, 0.4 µM of 5′ and 3′ primers and iQ-SYBR green supermix reagent (Bio-Rad). Gene expression was normalized to that of *GAPDH*. The primer sequences used for real-time PCR are listed in Table 1.

### 2.6. Statistical Analysis

The results were analysed using two-tailed Student’s *t*-test and expressed as mean ± standard error (SE). All statistical analyses were conducted using Prism 8 software version 8.0.2 for Windows (GraphPad, La Jolla, CA, USA). A *p*-value of less than 0.05 was considered statistically significant.

## 3. Results

### 3.1. Ischemia-Reperfusion Impaired Kidney Function and Altered Oxidative Stress Biomarkers in the Plasma

**Figure 1:**
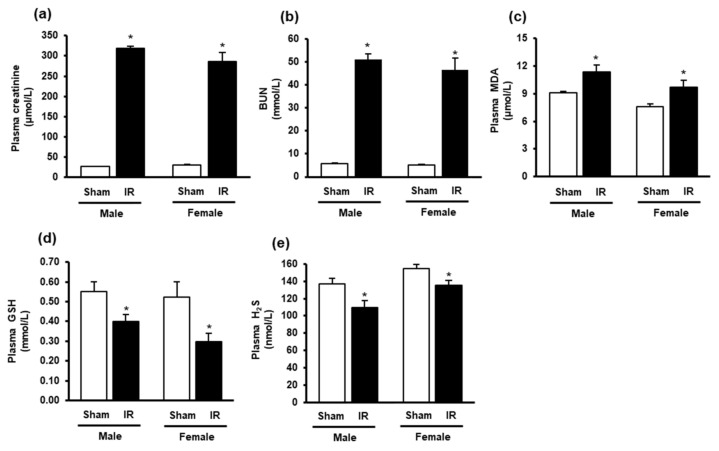
Plasma creatinine, blood urea nitrogen, malondialdehyde, glutathione and hydrogen sulfide levels in rats. The left kidney of male and female rats was subjected to 45 min ischemia followed by 24 h reperfusion (IR) or without ischemia (sham) as a control. The plasma (**a**) creatinine, (**b**) blood urea nitrogen (BUN), (**c**) malondialdehyde (MDA), (**d**) reduced glutathione (GSH) and (**e**) hydrogen sulfide (H_2_S) were measured. The results are expressed as mean ± SE (*n* = 5–6). * *p* < 0.05 when compared with the value obtained from the sham group.

Kidney ischemia-reperfusion caused a significant elevation of plasma creatinine and BUN levels in both male and female rats, compared to that in sham-operated rats (Figure 1a,b), indicating impaired kidney function. The plasma MDA levels were significantly elevated in rats 24 h after kidney ischemia-reperfusion (Figure 1c), indicating elevated lipid peroxidation. These rats had lower plasma GSH and H_2_S levels than the sham-operated rats (Figure 1d,e). The results suggested that rats subjected to ischemia-reperfusion developed AKI and altered oxidative stress biomarkers in the circulation.

### 3.2. Kidney Ischemia-Reperfusion Increased Lipid Peroxidation, Decreased Glutathione and Hydrogen Sulfide Levels in the Heart

**Figure 2:**
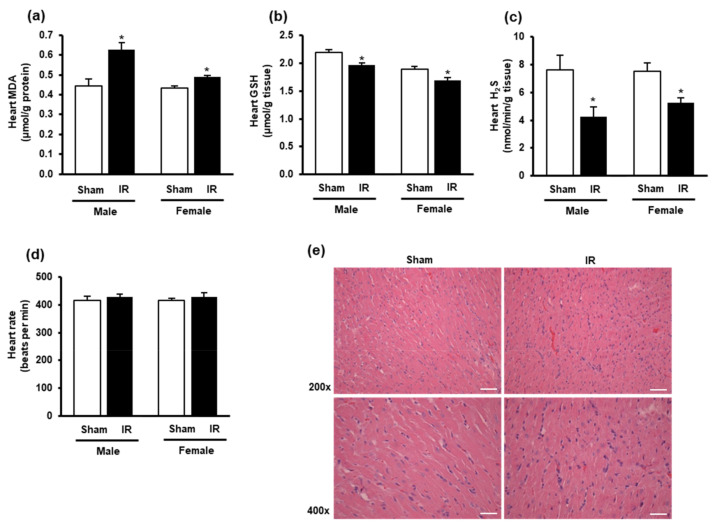
Lipid peroxidation, glutathione, hydrogen sulfide levels, heart rate, and histology in the heart. The left kidney of male and female rats was subjected to 45 min ischemia, followed by 24 h reperfusion (IR) or without ischemia (sham) as a control. The heart, (**a**) malondialdehyde (MDA), (**b**) reduced glutathione (GSH), (**c**) hydrogen sulfide (H_2_S), and (**d**) heart rate were measured. (**e**) The histological structure of the heart tissues was examined by hematoxylin and eosin (H and E) staining. The representative images (magnification 200×, 400×) are shown (scale bar = 100 μm). The results are expressed as mean ± SE (*n* = 4–6). * *p* < 0.05 when compared with the value obtained from the sham group.

The impact of kidney ischemia-reperfusion on the heart was examined by measuring oxidative stress biomarkers in the heart. There was a significant increase in lipid peroxidation in the hearts of both male and female rats at 24 h after kidney ischemia-reperfusion (Figure 2a), indicating an increase in oxidative stress in the heart. The GSH and H_2_S levels were significantly decreased in the heart of these rats (Figure 2b,c). There was no significant change in the heart rate (Figure 2d) nor histological change in the heart tissue after kidney ischemia-reperfusion (Figure 2e). The results suggested that kidney ischemia-reperfusion injury resulted in an increased oxidative stress in the heart.

### 3.3. Effect of Kidney Ischemia-Reperfusion on Glutathione and Hydrogen Sulfide Synthesizing Enzyme Expression in the Heart

**Figure 3:**
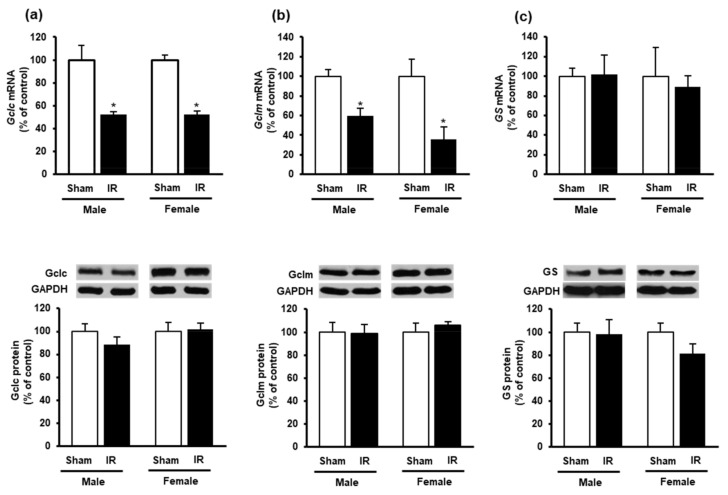
Effect of kidney ischemia-reperfusion on glutathione-synthesizing enzyme expression in the heart. The left kidney of male and female rats was subjected to 45 min ischemia followed by 24 h reperfusion (IR) or without ischemia (sham). The mRNA and protein expressions of (**a**) glutamate-cysteine ligase catalytic subunit (Gclc), (**b**) glutamate-cysteine ligase modifier subunit (Gclm) and (**c**) glutathione synthetase (GS) were measured in the hearts. The representative images of Western blots are shown. Results are expressed as mean ± SE (*n* = 4–6). * *p* < 0.05 when compared with the value obtained from the sham group.

To investigate if kidney ischemia-reperfusion affected GSH production in the heart, we examined the expression of GSH-synthesizing enzymes. The mRNA expression of glutamate-cysteine ligase (*Gclc* and *Gclm*) subunits were significantly reduced in the heart of both male and female rats after kidney ischemia-reperfusion as compared to the sham-operated group (Figure 3a,b). However, there was no significant change in Gclc and Gclm protein levels in the heart (Figure 3a,b). Furthermore, there was no significant change in glutathione synthetase mRNA or protein in the heart (Figure 3c).

**Figure 4:**
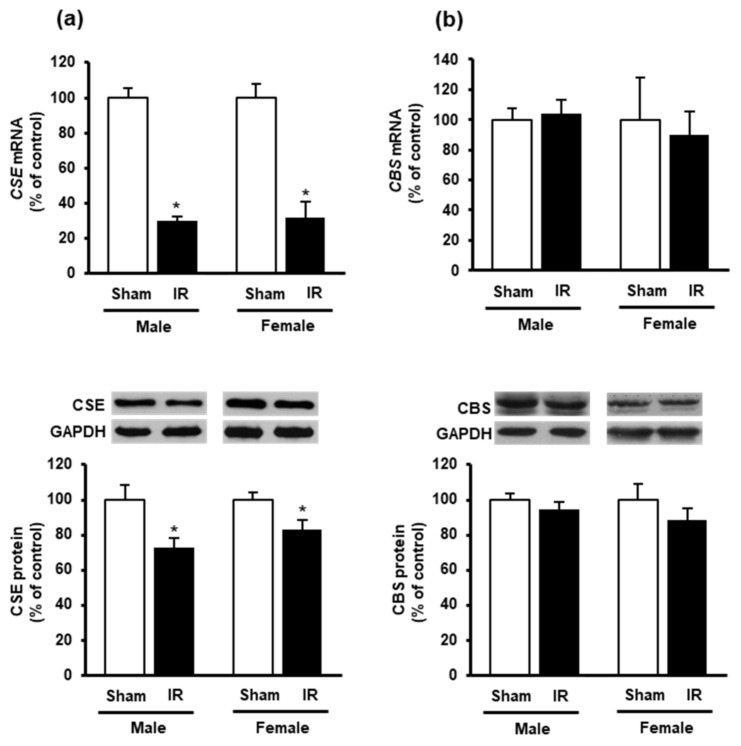
Effect of kidney ischemia-reperfusion on cystathionine γ-lyase and cystathionine β-synthase expression in the heart. The left kidney of male and female rats was subjected to 45 min ischemia followed by 24 h reperfusion (IR) or without ischemia (sham) as a control. The mRNA and protein expressions of (**a**) cystathionine γ-lyase (CSE) and (**b**) cystathionine β-synthase (CBS) were measured in the hearts. The representative images of Western blots are shown. Results are expressed as mean ± SE (*n* = 4–6). * *p* < 0.05 when compared with the value obtained from the sham group.

To investigate if kidney ischemia-reperfusion could affect H_2_S production in the heart, we examined the expression of H_2_S-synthesizing enzymes CBS and CSE. Kidney ischemia-reperfusion caused a significant reduction of CSE mRNA and protein levels in the heart of both male and female rats (Figure 4a), while did not alter CBS expression (Figure 4b).

**Figure 5:**
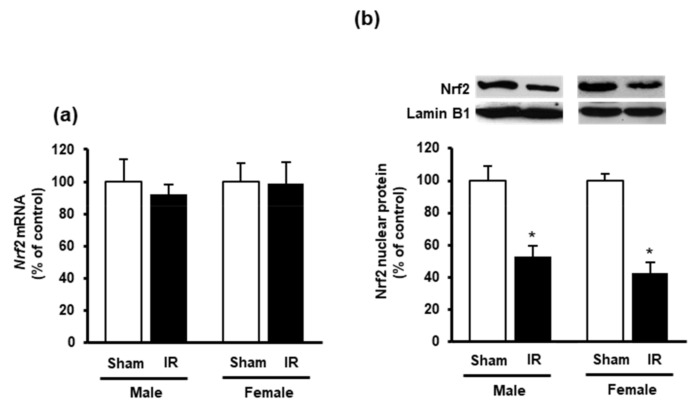
Effect of kidney ischemia-reperfusion on Nrf2 expression in the heart. The left kidney of male and female rats was subjected to 45 min ischemia followed by 24 h reperfusion (IR) or without ischemia (sham). (**a**) *Nrf2* mRNA of the heart was measured using RT-qPCR analysis. (**b**) The Nrf2 protein in the nucleus was determined by Western immunoblotting analysis. The representative images of Western blots are shown. Results are expressed as mean ± SE (*n* = 4). * *p* < 0.05 when compared with the value obtained from the sham group.

**Figure 6:**
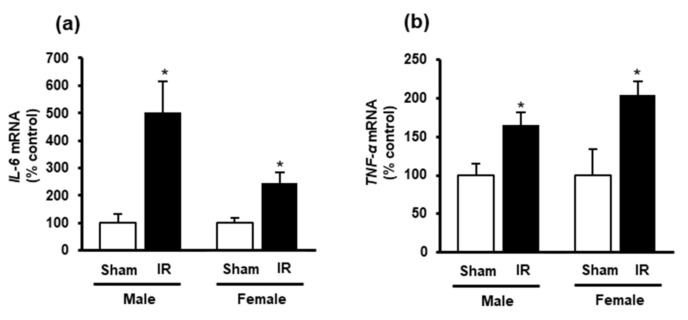
Effect of kidney ischemia-reperfusion on IL-6 and TNF-α expression in the heart. The left kidney of male and female rats was subjected to 45 min ischemia followed by 24 h reperfusion (IR) or without ischemia (sham) as a control. (**a**) Interleukin-6 *(IL-6*) and (**b**) tumor necrosis factor-α (*TNF-α*) mRNA was measured in the hearts. The results are expressed as mean ± SE (*n* = 5–6). * *p* < 0.05, when compared with the value obtained from the sham group.

Next, we measured the nuclear Nrf2 in the heart as its translocation from the cytoplasm into the nucleus is an essential step for the upregulation of antioxidant enzyme gene expression. Western immunoblotting analysis of nuclear proteins showed a significant reduction of Nrf2 in the heart of rats subjected to kidney ischemia-reperfusion injury compared to the sham-operated group (Figure 5b). There was no significant change of *Nrf2* mRNA expression in the heart (Figure 5a). These results suggested that kidney ischemia-reperfusion could diminish CSE-mediated H_2_S synthesis and reduce nuclear Nrf2, leading to an increased oxidative stress in the heart. Furthermore, a significant elevation of inflammatory cytokines (*IL-6* and *TNF-α*) was observed in the heart upon kidney ischemia-reperfusion injury (Figure 6a,b).

## 4. Discussion

Although AKI is known to lead to adverse cardiovascular outcomes, the underlying mechanisms are not well-understood. In the present study, kidney ischemia-reperfusion injury caused a significant decrease in antioxidant molecules (GSH and H_2_S) in the heart, as well as in the plasma. Our results, for the first time, demonstrated that ischemia-reperfusion-induced AKI could inhibit the expression of H_2_S-synthesizing enzyme (CSE) in the heart. A reduction of endogenous H_2_S synthesis, might weaken the Nrf2-mediated antioxidant defense mechanism and lead to increased cardiac oxidative stress. Male and female rats did not exhibit a significant difference for any of the parameters measured.

Overproduction of ROS and/or impaired antioxidant defense can lead to increased oxidative stress [8,32]. Studies have shown that excessive ROS can alter iron channels, vasomotor function and cytokine expression, which, in turn, exert detrimental effects to the heart [33,34]. Previous studies reported myocardial injury with increased oxidative stress and inflammatory cytokines in the rat heart at 3 h to 4 h after kidney ischemia-reperfusion [35,36,37]. In the present study, increased oxidative stress and inflammatory cytokine expression (IL-6 and TNF-α) were still detectable in the heart at 24 h after kidney ischemia-reperfusion injury. These results indicated that cardiac oxidative stress was not a transient response to AKI. A prolonged exposure of the heart to oxidative stress may further impair cardiac function.

Glutathione is a major endogenous non-enzymatic antioxidant. Its depletion directly correlates with an increase in oxidative stress. More than 90% of endogenous glutathione is in its reduced form (GSH) [9,10]. A previous study reported that lower cardiac GSH levels increased the susceptibility and the extent of myocardial injury [38]. Glutathione deficiency was linked to functional and structural abnormalities in the heart of patients with coronary artery disease or terminal cardiomyopathy [39]. Another study reported that the supplementation of GSH improved cardiac mechanical function in rats with decreased myocardial GSH levels following cardiac ischemia-reperfusion [40]. In the present study, a decrease in GSH levels was detected in the heart and plasma of rats at 24 h after kidney ischemia-reperfusion injury. A reduction of GSH was accompanied by an increase in lipid peroxidation in the heart and plasma, indicating oxidative stress. These results were in line with a metabolomic assessment that showed a decrease of GSH level in the rat heart 24 h after kidney ischemia-reperfusion injury [41]. However, no mechanism was proposed. The heart is capable of synthesizing GSH by glutamate-cysteine ligase and glutathione synthetase [42]. Although, we observed a reduction of glutamate-cysteine ligase (*Gclc, Gclm*) mRNA expression in the heart at 24 h after kidney ischemia-reperfusion, their protein levels were not significantly changed in the heart nor any change in glutathione synthetase expression. We speculated that a decreased GSH level in the heart might be a consequence of its increased utilization to combat oxidative stress and/or decreased supply from the circulation. Alternatively, a reduction of cardiac glutamate-cysteine ligase (Gclc, Gclm) protein levels and/or glutathione synthetase expression might occur at later time points after kidney ischemia-reperfusion, which in turn, could reduce GSH production in the heart.

Aside from glutathione, H_2_S is another biomolecule that exerts cardiovascular protective effect through redox balance and vessel relaxation [19,23]. In the present study, kidney ischemia-reperfusion caused a significant reduction of H_2_S levels in the heart. CSE is the major enzyme responsible for H_2_S synthesis in the cardiovascular system [15]. Kidney ischemia-reperfusion decreased both gene and protein expression of CSE, suggesting that AKI could inhibit H_2_S synthesis in the heart. Previous studies conducted in our laboratory and others showed a significant decrease in renal CSE and CBS expression upon kidney ischemia-reperfusion [26,28,43]. We also observed a decrease in CSE expression in the liver upon kidney ischemia-reperfusion [29]. Taken together, these results suggest that kidney ischemia-reperfusion impairs the expression of H_2_S-synthesizing enzymes in the kidney as well as in distant organs (heart and liver). It was reported that injection of H_2_S donors could preserve mitochondrial function and reduce cardiac infarct size in mice [15]. Exogenous H_2_S donor could also up-regulate endothelial nitric oxide synthase-mediated nitric oxide bioavailability against heart failure [17]. Furthermore, administration of sodium hydrogen sulfide significantly improved the cardiac antioxidant defense by increasing the GSH levels during myocardial ischemia injury [44]. Our previous study reported that administration of H_2_S donor (sodium hydrogen sulfide) improved kidney function in rats with kidney ischemia-reperfusion injury [27]. However, it remains to be investigated if restoration of endogenous H_2_S synthesis or supplementation of H_2_S donors can attenuate oxidative stress in the heart upon kidney ischemia-reperfusion injury. Another mechanism by which H_2_S may exert the antioxidant effect is through its interaction with the Nrf2 antioxidant signaling pathway [45]. Nrf2 plays an important role in regulating the expression of antioxidant enzymes particularly the phase II antioxidant enzymes, such as glutamate-cysteine ligase (Gclc, Gclm), glutathione peroxidase, glutathione S-transferase, heme oxygenase-1 [46]. Under unstressed conditions, Nrf2 is kept in the cytoplasm of the cell through binding to kelch-like ECH-associated protein 1 (Keap1) [20]. Increased oxidative stress leads to Nrf2 disassociates from Keap1, and translocates to the nucleus where it binds to the antioxidant response element (ARE) and upregulates the gene expression of antioxidant enzymes [20]. The H_2_S-induced S-sulfhydration of Keap1 leads to Nrf2 dissociation and translocation into the nucleus, which in turn, activates Nrf2 transcriptional regulation of antioxidant genes [47,48]. In the present study, the level of nuclear Nrf2 protein in the heart was significantly decreased upon kidney ischemia-reperfusion while *Nrf2* mRNA was not changed. We speculate that low level of H_2_S might contribute to less Nrf2 being translocated into the nucleus. On the other hand, the lack of Nrf2 upregulation might dampen its positive effect on the antioxidant defense upon kidney ischemia-reperfusion.

The strengths and limitations of the present study should be considered. To our knowledge, this is the first study to suggest that kidney ischemia-reperfusion can cause a significant decrease in cardiac CSE expression and H_2_S synthesis in the heart of male and female rats. There is an increased recognition that sex differences exist in cardiovascular diseases [49,50,51]. However, the sex impact of AKI on cardiovascular function remains to be investigated. In the present study, we did not observe a significant difference in AKI-induced cardiac oxidative stress in male and female rats at 24 h after kidney ischemia-reperfusion. It remains to be investigated if differential effect occurs at later time points after kidney ischemia-reperfusion injury. Low levels of H_2_S can lead to downregulation of the Nrf2 signaling pathway and increased cardiac oxidative stress. However, the present study did not have direct evidence on a causal relationship between AKI-impaired H_2_S synthesis and downregulation of Nrf2 in the heart. Previous studies documented functional and structural abnormalities in the heart at 3 h or 72 h after kidney ischemia-reperfusion in male rats [35,41]. Since the low level of H_2_S has been shown to associate with increased risk of cardiovascular disease [12], future studies are warranted to investigate if a restoration of CSE-mediated H_2_S synthesis or supplementation of exogeneous H_2_S donors can attenuate AKI-induced oxidative stress in the heart and improve cardiac function.

## 5. Conclusions

The present study has demonstrated that kidney ischemia-reperfusion injury elicits oxidative stress in the heart as indicated by increased lipid peroxidation and decreased antioxidant defense (decreased GSH, H_2_S and Nrf2), as well as increased inflammatory cytokine expression. Our results suggest that the inhibition of CSE-mediated H_2_S synthesis upon kidney ischemia-reperfusion may lead to the downregulation of Nrf2-mediated antioxidant defense and increased oxidative stress and inflammation in the heart. This, in turn, contributes to increased risk of cardiovascular disease in AKI. Future studies are warranted to confirm (1) a causal relationship between a low level of H_2_S synthesis and a reduced Nrf2 activation; and (2) if restoration of H_2_S can activate Nrf2 and increase antioxidant defense.

## Figures and Tables

**Table 1:** Primer sequences used for real-time PCR.

Target Gene	Forward Primer (5′–3′)	Reverse Primer (5′–3′)	Accession Number	Size (bp)
*CSE*	GTTGGGTTTGTGGGTGTTTC	GTATGGAGGCACCAACAGGT	XM_008761574.2	150
*CBS*	TCGTGATGCCAGAGAAGATG	TTGGGGATTTCGTTCTTCAG	NM_012522.2	148
*Gclc*	GCCCAATTGTTATGGCTTTG	AGTCCTCTCTCCTCCCGTGT	NM_012815.2	124
*Gclm*	CGAGGAGCTTCGAGACTGTAT	ACTGCATGGGACATGGTACA	NM_017305.2	114
*GS*	ACAACGAGCGAGTTGGGAT	TGAGGGGAAGAGCGTGAATG	NM_012962.1	182
*IL-6*	CCGGAGAGGAGACTTCACAG	ACAGTGCATCATCGCTGTTC	NM_012589.2	161
*TNF-α* *Nrf2*	CCCAGACCCTCACACTCAGAT CTGTCAGCTACTCCCAGGTTG	TTGTCCCTTGAAGAGAACCTG GCGACTCATGGTCATCTACAA	XM_008772775.2 NM_031789.2	215 111
*GAPDH*	AGAGAAGGCAGCCCTGGT	GCTCTCTGCTCCTCCCTGT	NM_017008.4	138
